# Observation on the Clinical Effect of Apatinib Combined with Chemotherapy in the Treatment of Advanced Non-Small Cell Lung Cancer

**DOI:** 10.12669/pjms.37.4.4066

**Published:** 2021

**Authors:** Yu-jie Cui, Jia Liu, Miao-miao Liu, Hong-zhen Zhang

**Affiliations:** 1 Yu-jie Cui, Department of Oncology, Hebei General Hospital, Shijiazhuang, 050051, Hebei, China; 2 Jia Liu, Department of Hematology, Affiliated Hospital of Hebei University, Baoding, 071000, Hebei, China; 3 Miao-miao Liu, Department of Oncology, Hebei General Hospital, Shijiazhuang, 050051, Hebei, China; 4 Hong-zhen Zhang, Department of Oncology, Hebei General Hospital, Shijiazhuang, 050051, Hebei, China

**Keywords:** apatinib, chemotherapy, advanced NSCLC, treatment

## Abstract

**Objectives:**

To evaluate the clinical effect of apatinib combined with chemotherapy in the treatment of advanced non-small cell lung cancer (NSCLC).

**Methods:**

Eighty patients with advanced NSCLC treated in Hebei General Hospital from January 2017 to July 2020 were randomly divided into two groups: the experimental group and the control group, each with 40 cases. Patients in the control group were treated with conventional paclitaxel combined with cisplatin chemotherapy, while patients in the experimental group were treated with apatinib mesylate tablets based on the treatment of the control group. After treatment, tumor efficacy evaluation was conducted on all patients every two cycles, and the therapeutic effect, adverse drug reactions, improvement of quality-of-life scores prior to and after treatment, and changes of indicators such as tumor markers carcinoembryonic antigen (CEA) and carbohydrate antigen 153(CA153) were compared and analyzed between the two groups.

**Results:**

The total effective rate of the experimental group was 67.5%, which was significantly better than the 45% of the control group (p=0.04); The incidence of adverse drug reactions in the experimental group was 25%, while that in the control group was 37.5%, with no significant difference (p=0.23); Moreover, the improvement rate of quality of life scores in the experimental group was significantly higher than that in the control group (p=0.03), and the levels of CEA and CA153 in the experimental group were significantly lower after treatment than those in the control group, with a statistically significant difference (p=0.01).

**Conclusion:**

Apatinib combined with conventional chemotherapy is effective in the treatment of advanced non-small cell lung cancer, the quality of life can be significantly improved, tumor markers can be significantly reduced, and adverse reactions will not be significantly increased.

## INTRODUCTION

Lung cancer is the leading cause of cancer death worldwide.^1^ Especially non-small cell lung cancer (NSCLC), as one of the common types of lung cancer, accounts for about 80%-85% of lung cancer, and its incidence rate has increased in recent years, causing serious impact on the health and life of patients.^2^ NSCLC occurs under the action of a variety of factors, with its known inductors including smoking, bad living habits, heredity, etc., most of which have no specific clinical manifestations in the early stage, and present symptoms of cough and sputum. Consequently, most of the confirmed NSCLC cases found in the clinical diagnosis work are at an advanced stage, and the opportunity for surgical treatment is lost, causing a fatal blow to the prognosis of patients.^3^ Chemotherapy is commonly used as palliative treatment for patients with intermediate and advanced NSCLC. It can kill or inhibit tumor cells by chemotherapy drugs to achieve clinical treatment purposes. Despite that prognosis and survival rate can be improved with chemotherapy, adverse reactions are induced accordingly, which has a bearing on the pathological characteristics and epidermal growth factor receptor (EGFR) expression of patients.^4^ Evidence shows that targeted therapy is superior to traditional cytotoxic chemotherapy in terms of clinical effects in the treatment of advanced NSCLC.^5^

A certain effect has been achieved by oral administration of apatinib mesylate tablets combined with conventional chemotherapy (paclitaxel and cisplatin) in the treatment of advanced NSCLC. Details are reported as follows.

## METHODS

### Ethical Approval

The study was approved by the Institutional Ethics Committee of Hebei General Hospital (No. 2017-181) on January 7, 2018, and written informed consent was obtained from all participants.

### Inclusion criteria:

1. NSCLC patients with EGFR wild-type confirmed by puncture pathological examination.^6^
2. Advanced stage patients (clinical stage ≥ Phase III).^7^
3. Patients with clear lesions on chest Computed tomography (CT) or Magnetic resonance imaging (MRI) and can be accurately measured^8^.
4. Patients with Karnofsky Performance Status (KPS) score > 80.
5. Patients whose family members are willing and able to cooperate to complete the study and have good treatment compliance.
6. Patients who have no contraindications to the drugs used in this study.
7. Patients who signed the informed consent.

### Exclusion criteria:

1. Patients with poor physical quality, cachexia and unstable vital signs.
2. Patients complicated with other systemic malignancies.
3. Patients who are allergic or contraindicated to the medication involved in the study.
4. Patients complicated with severe organic or congenital diseases of heart, liver and kidney.
5. Patients with mental illness who cannot cooperate to complete the study.
6. Patients who have recently taken relevant drugs such as immunosuppressants and hormones that affect the study in the near future.

Eighty patients with advanced NSCLC admitted to Hebei General Hospital from January 2017 to July 2020 were randomly divided into two groups, with 40 cases in each group. Twenty-six males and 14 females were grouped into the experimental group, aged 43-75 years old, with an average of 61.00±9.88 years old. 25 males and 15 females were grouped into the control group, aged 41-74 years old, with an average of 61.11±8.57 years old. There was no significant difference in general data between the two groups, which were comparable (Table-I).

**Table-I:** Comparative analysis of general data between experimental group and control group (*X̅* ±S) n=40.

Indicators	Experimental group	Control group	t/χ^2^	p
Age	61.00±9.88	61.11±8.57	0.05	0.96
Male (%)	26(65%)	25(62.5%)	0.05	0.82
***Pathological type***				
Adenocarcinoma	23(57.5%)	22(55%)	0.05	0.82
Squamous cell carcinoma	14(35%)	16(40%)	0.21	0.64
Other types	3(7.5%)	2(5%)	0.23	0.61
***Tumor location***				
Peripheral type	27(67.5%)	25(62.5%)	0.83	0.36
Central type	13(32.5%)	15(37.5%)	0.73	0.26
Clinical stage				
III	28(70%)	30(75%)	0.25	0.62
IV	12(30%)	10(25%)	0.27	0.63

p>0.05.

### Treatment methods

Both groups of patients underwent blood cell analysis, liver function, and renal function tests, and the abnormal indicators were corrected accordingly. Hydration was performed one day before chemotherapy, and the conventional chemotherapy regimen of paclitaxel combined with cisplatin was adopted by the control group: paclitaxel 75-145mg/m2 and cisplatin 25mg/m2 for more than two hour. During chemotherapy, patients in the control group were tested and treated with antiemesis, protection of liver and kidney function, and fluid replenishment, etc., with three weeks as one course of treatment. In contrast, the experimental group was given apatinib mesylate tablets at 500mg/d for three weeks on the basis of the treatment regimen of the control group.

### Observation indicators:

### 1) Efficacy evaluation

All patients underwent tumor efficacy evaluation every two cycles after treatment for three consecutive months. Efficacy judgment: based on the standards established by World Health Organization (WHO)^9^: Complete remission (CR): the mass disappears completely, lasting for ≥4 weeks; Partial remission (PR): the product of the two greatest diameters of the tumor reduces by > 50% compared with that before treatment, lasting for ≥ 4 weeks; Stable disease (SD): the product of the two greatest diameters of the mass decreases by < 5% and increases by ≤ 25%, lasting for ≤ 4 weeks; Progression disease (PD): the product of the two greatest diameters of the mass increases by 25% or a new lesion appears. The total effective rate is calculated by CR and PR.

### 2) Adverse drug reaction evaluation

Adverse drug reactions of the two groups occurred within one month after medication were recorded, including proteinuria, leucopenia, erythrocytopenia, thrombocytopenia, gastrointestinal symptoms and other adverse reactions.

### 3) Quality of life score

ECOG score^10^ was used to observe the improvement of quality of life before and after treatment: improvement (score reduction ≥ 1 point), stable (score unchanged), deterioration (score increase ≥ 1 point).

### 4) Comparative analysis of tumor markers

early morning fasting blood was drawn before treatment and after 1 month of treatment to detect tumor markers such as CEA and CA153, and the differences between the two groups were compared and analyzed.

### Statistical Analysis

All the data were statistically analyzed by SPSS 20.0 software, and the measurement data were expressed as (±s). Two independent sample t-test was used for inter-group data analysis, paired t test was used for intra-group data analysis, and 2-test was adopted for rate comparison. P<0.05 indicates a statistically significant difference.

## RESULTS

The comparative analysis of the treatment effect of the two groups is shown in Table-II, suggesting that the total effective rate of the experimental group is 67.5% after treatment, which was significantly better than the 45% of the control group, with a statistically significant difference (p=0.04)

**Table-II:** Comparative analysis of treatment effect between the two groups (*X̅* ±S) n=40.

Group	CR	PR	SD	PD	Total effective rate
Experimental group	14	13	11	2	27(67.5%)
Control group	8	10	12	10	18(45%)
χ^2^					4.11
p					0.04

p<0.05.

The incidence of adverse drug reactions in the two groups after treatment was compared and analyzed, suggesting that the incidence of adverse reactions in the experimental group was 25%, and that in the control group was 37.5%. There was no significant difference in the incidence of adverse reactions between the two groups (p=0.23). (Table-III)

**Table-III:** Comparative analysis of adverse drug reactions of the two groups after treatment (*X̅* ±S) n=40.

Group	Proteinuria	WBC reduction	RBC reduction	PLT reduction	Gastrointestinal reaction	Incidence rate
Experimental group	1	3	2	0	4	10(25%)
Control group	0	2	3	6	4	15(37.5%)
χ^2^						1.45
p						0.23

p<0.05.

After treatment, the improvement rate of quality of life score in the experimental group was significantly higher than that in the control group (p=0.03), and the experimental group boasted a more obvious advantage in the quality of life score (Table-IV).

**Table-IV:** Comparative analysis of quality of life score (ECOG) between the two groups prior to and after treatment (*X̅* ±S) n=40.

Group	Improvement*	Stable	Deterioration
Experimental group	23	11	6
Control group	13	16	11
χ^2^	5.05	1.39	1.86
p	0.03	0.24	0.17

* p<0.05.

Prior to treatment, the levels of CEA and CA153 in the two groups were significantly higher than those in the normal group, but no significant difference could be found between the levels of CEA and CA153 between the two groups (p>0.05). The above indicators decreased after treatment, with statistically significant differences (p<0.05). After treatment, CEA and CA153 in the experimental group were significantly lower than those in the control group, with statistically significant differences (p=0.01) (Table-V)

**Table-V:** Comparative analysis of tumor marker levels between the two groups prior to and after treatment (*X̅* ±S) n=40.

	CEA (ng/ml)				CA153(U/mL)			

Group	Prior to treatment*	After treatmentΔ	t	p	Prior to treatment*	After treatmentΔ	t	p
Experimental groupΔ	96.14±30.73	27.74±8.61	13.55	0.00	74.28±13.65	24.52±4.77	21.77	0.00
Control groupΔ	97.63±28.41	36.58±8.49	13.02	0.00	76.29±12.83	35.61±6.83	17.70	0.00
t	0.23	4.62			0.68	8.42		
p	0.82	0.00			0.50	0.00		

* p>0.05, Δp<0.05.

## DISCUSSION

NSCLC is a common type of lung cancer in clinical practice, accounting for over 80% of the incidence of lung cancer, among which squamous cell carcinoma and adenocarcinoma are common.^11^ In recent years, the incidence of NSCLC has been increasing year by year, which is an important malignant tumor leading to death. NSCLC is easy to be ignored due to the lack of specific clinical symptoms in the intermediate and advanced stage, so it is mostly found in the middle and late stage, when the opportunity for surgical treatment has been lost. Clinically, chemotherapy is often used to control the growth of tumor cells, prolong the life span of patients and improve the quality of life of patients. Despite that patients with advanced NSCLC have received standard chemotherapy and radiotherapy with clear curative purpose for many years, most of them have suffered rapid disease progression and poor prognosis.^12^

With the deepening of tumor pathogenesis, traditional cytotoxic chemotherapy and radiotherapy may have reached a stable stage, and the selection of integrated molecular targeted drugs and immunotherapy in the future may have a broader prospect. It is believed by Dafni et al.^13^ that the occurrence of NSCLC is the result of the synergistic effect of multiple factors, and higher efficacy is shown by multi-target therapy for patients with advanced NSCLC than chemotherapy alone or any other monotherapy. With the continuous development of immunology and molecular biology, great changes have taken place in the treatment of advanced NSCLC.^14^ It is considered by Valentino et al.^15^ that the level of vascular endothelial growth factor (VEGF) in patients with NSCLC is so high that it binds to vascular endothelial growth factor receptor 2 (VEGFR-2), resulting in increased number of tumor neovasculature, enhanced vascular permeability, increased endothelial cell viability, and promoted chemotaxis and metastasis of tumor cells via complex regulatory mechanisms.^16^ Therefore, antiangiogenic therapy is the key to the treatment of lung cancer.^17^

Apatinib mesylate, as a targeted anti-angiogenesis drug, exerts its unique role in not only effectively inhibiting the activation of VEGFR-2 tyrosine kinase, but also hindering the growth and proliferation of vascular endothelial cells, thus forming its anti-tumor effect. According to Zhang et al.^18^, apatinib is effective in the treatment of patients with EGFR-positive advanced NSCLC. It is considered by Gandhi^19^ for patients with EGFR-negative metastatic NSCLC who have not been treated previously, the combination of platinum chemotherapy drugs can also significantly increase the treatment effect of patients. It is confirmed in our study that the total effective rate of patients receiving apatinib combined chemotherapy after treatment was 67.5%, while that of those who receiving chemotherapy alone was 45%, with a statistically significant difference (p=0.04), suggesting that more favorable treatment effects could be obtained by apatinib combined with chemotherapy. A meta-analysis by Yu suggested that the combination of apatinib and chemotherapy for advanced NSCLC did not significantly increase toxicity.^20^ Meanwhile, Liu’s report in the same period suggested that apatinib had partial reactions but only mild side effects, including mild hypertension, vomiting and hand-foot syndrome, which could be controlled.^21^ According to our study, there was no significant difference in the incidence of adverse drug reactions between the two groups (p=0.23), which also confirmed that the combination of apatinib and chemotherapy did not increase adverse reactions.

Total 915 patients with advanced NSCLC were recruited into a meta-analysis by Zhang et al.^22^ for 5 Phase II/III randomized trials. MTC shows that the choice of apatinib in advanced NSCLC can reduce the dose of chemotherapy drugs, thereby reducing the adverse reactions of chemotherapy drugs, and can increase the tolerance and quality of life of patients. According to the study conducted by Zhang and colleagues^23^, with the combination of apatinib mesylate, a better therapeutic effect has been achieved in intermediate and advanced NSCLC, with improved immune resistance, reduced tumor marker level, and less side effects. Li et al.^24^ has demonstrated that apatinib combined with first-line chemotherapy in patients with advanced NSCLC was safe and beneficial for extending median survival. Zhang et al^25^ have also suggested that apatinib combined with vinorelbine may be an effective and safe follow-up therapy for patients with advanced NSCLC. It has also been confirmed by our study that the improvement rate of quality-of-life score in the experimental group was significantly higher than that in the control group (p=0.03), and CEA and CA153 in the experimental group were significantly lower than those in the control group (p=0.01).

### Limitations of the Study

The sample size is small and the follow-up time is short; only one overall study was conducted on patients with NSCLC due to sample size problems, but there was no more precise distinction for their pathological subtypes. Active countermeasures and solutions are being adopted: further collection of data and increase of follow-up time; Further refinement of more precise research based on the pathological subtypes of NSCLC is suggested. Further confirmation of the therapeutic effect and long-term effect of apatinib in NSCLC patients with different pathological subtypes.

## CONCLUSION

Apatinib combined with conventional chemotherapy has proved to be effective in the treatment of advanced NSCLC. With such a safe and effective treatment regimen, the quality of life was significantly improved, tumor markers were significantly reduced, and no significant increase was seen in adverse reactions.

### Authors’ Contributions:

**YJC** & **JL:** Designed this study and prepared this manuscript, and are responsible and accountable for the accuracy or integrity of the work.

**HZZ:** Collected and analyzed clinical data.

**MML:** Significantly revised this manuscript.
